# Osteoporosis Knowledge, Self-Efficacy, and Beliefs among College Students in the USA and China

**DOI:** 10.4061/2011/729219

**Published:** 2011-04-26

**Authors:** M. Allison Ford, Martha Bass, Yan Zhao, Jin-Bing Bai, Yue Zhao

**Affiliations:** ^1^Bone Density Laboratory, The University of Mississippi, 222 Turner Center, Oxford, MS 38677, USA; ^2^Medical-Surgical Department, School of Nursing, Tianjin Medical University, 22 Qi Xiang Tai Road, He Ping District, P.O. Box 300070, Tianjin, China

## Abstract

This study investigated differences in osteoporosis knowledge, self-efficacy, and health beliefs among Chinese and American college students. Information obtained will be used in developing osteoporosis prevention programs for younger adults. *Methods*. Chinese (*n* = 409) and US (*n* = 408) college students completed the Osteoporosis Health Belief, Self-Efficacy, and Knowledge Tests. *Results*. Differences were seen in osteoporosis knowledge (*M*_us_ = 14.52, *M*_Chinese_ = 11.82), exercise knowledge (*M*_us_ = 8.16, *M*_Chinese_ = 9.04), calcium knowledge (*M*_us_ = 8.47, *M*_Chinese_ = 9.73), perceptions of exercise benefits (*M*_us_ = 24.07, *M*_Chinese_ = 21.09), calcium benefits (*M*_us_ = 23.17, *M*_Chinese_ = 18.36), exercise barriers (*M*_us_ = 11.75, *M*_Chinese_ = 14.96), calcium barriers (*M*_us_ = 13.04, *M*_Chinese_ = 15), and exercise self-efficacy (*M*_us_ = 73.71, *M*_Chinese_ = 63.81). *Conclusion*. US college students know more about osteoporosis and its risk factors; however, there are similarities in perception of risk between US and Chinese students. Chinese students perceive greater barriers to reducing their risk through exercise and dietary calcium intake.

## 1. Introduction

Osteoporosis is a significant global public health issue, expected to affect more people worldwide than ever by 2050 [1]. In the United States, it has been estimated that by the end of 2010, approximately 12 million people over the age of 50 years will have osteoporosis with another 40 million being osteopenic. These numbers are expected to increase to 14 million cases of osteoporosis and over 47 million cases of low bone mass in 2020 [2]. This increase may cause the number of hip fractures to triple by 2040 [3]. Annual direct care cost for osteoporotic fractures range from approximately $12 to $18 billion per year, while indirect costs are likely in the billions of dollars [2]. Furthermore, it is projected that that these costs could double or triple in the coming decades [2, 3]. As reported in the U.S. Surgeon General's Report on Bone Health and Osteoporosis, by 2020, 50% of Americans over the age of 50 will be at an increased risk for fractures from osteoporosis and low bone mass without direct and immediate prevention [3].

As noted, osteoporosis is a global concern with an estimated 9 million osteoporotic fractures worldwide for which Europe and the Americas accounted for 51% of all fractures, while most of the remainder occurred in the Western Pacific region and Southeast Asia [4]. According to the International Osteoporosis Foundation, more than half of the world's cases of fractures due to decreased bone mass will be in Asia by 2050. This assertion identifies the growing concern that with the aging population, longer life expectancy, and many dramatic life style changes in recent years, the prevalence of osteoporosis in China is likely to increase in the near future [5].

Compared to the United States, China is one of the most populous countries in the world. This significant growth in population may lead to a larger number of older adults and is a primary reason practitioners suggest osteoporosis is becoming more prevalent and will continue to increase throughout China. While the prevalence of low bone mass in China is still lower than in other industrialized countries, current statistics indicate the overall frequency of osteoporosis in mainland China is nearly 7% among all adults, 22.5% among men aged 50 years or older, and 40.1% among women aged 50 years or older [5]. These statistics are comparable to statistics in the United States in the late 80's and early 90's. These statistics draw a unique parallel between the historical significance of osteoporosis in the United States and the potential development of a similar epidemic in China. Furthermore, as indicated by The U.S. Surgeon General's Report on Bone Health and Osteoporosis, this parallel indicates the importance of the immediacy of prevention targeting the growing concerns in these two countries. 

As it is with a preponderance of chronic diseases, prevention is a key component to reducing the incidence rates of such diseases. This is indeed the case with osteoporosis and low-bone-mass-related diseases. A key component in developing successful prevention programs is the understanding and knowledge the population has about the disease [6]. More specifically related to low bone mass is the knowledge of dietary, exercise, and lifestyle characteristics that play a role in the development of osteoporosis. In understanding a populations knowledge, or lack thereof, practitioners can develop intervention strategies to address specific concerns across a myriad of populations. This is particularly important in college age individuals who are capable of making behavioral choices affecting some modifiable lifestyle changes including but not limited to low calcium intake, lack of physical activity, smoking, and alcohol consumption. Changing lifestyle and health behaviors at an early age will have a greater impact on the prevalence of osteoporosis and other chronic diseases.

In the USA, several studies have reported lack of knowledge about risk factors and preventative techniques in osteoporosis [6–9], however comparable studies on osteoporosis knowledge of Chinese are limited. In addition, the comparable Chinese studies have focused on the adult population and not that of college age individuals. Ip et al. [10] assessed awareness of osteoporosis among physicians in China and reported that 33% of physicians surveyed did not know that there were published guidelines for bone mineral density (BMD), 76% reported treating about three patients per week for osteoporosis, and 50% believed that an increase in BMD was important in prevention and treatment of the disease [10]. Ho et al. [11] suggested that a higher education level is associated with healthier diets and lower cardiovascular risk. The authors inferred that better educated individuals likely have better health knowledge and associated behaviors. As such the importance of understanding health knowledge related to osteoporosis is clear when evaluating current statistics in both the United States and developing countries. 

The purpose of this study was to (1) assess beliefs related to exercise and calcium intake, (2) measure osteoporosis self-efficacy, and (3) measure osteoporosis knowledge in college students in China and the US. With cultural, educational, and dietary differences, it is important to assess where differences lie for development of osteoporosis prevention programs.

## 2. Materials and Methods

### 2.1. Instrument

The Osteoporosis Health Belief, Self-Efficacy, and Knowledge Tests [12] were administered to undergraduate students (*N* = 817). These three instruments were designed to assess osteoporosis self-care during the early developmental stages. 

Based on the Health Belief Model (HBM), the Osteoporosis Health Belief Scale (OHBS) investigates beliefs associated with exercise and calcium intake. This scale was scored using a 5-point Likert scale ranging from “strongly disagree” to “strongly agree.” The 42 items on the OHBS are divided into 7 subscales of 6 items each. The subscales include susceptibility, seriousness, benefits of exercise, benefits of calcium intake, barriers to exercise, barriers to calcium intake, and health motivation. 

Drawing from the HBM, the *susceptibility* subscale measures an individual's perceived risk of developing osteoporosis. Questions assess the participants' perception of body build and family history as factors influencing their risk for osteoporosis as well as their chances of developing the disease. Questions regarding *seriousness* assess the perceived threat that osteoporosis presents to one's physical health, ability to complete daily tasks, and social status. How feelings towards oneself would change, seriousness of the disease, would you be crippled, financial expense, and fear of developing osteoporosis are assessed in this subscale. *Benefits of exercise* and *calcium intake* subscales assess belief in the efficacy of specific behaviors for preventing the occurrence of osteoporosis. 

Exercise questions focus on the preventive ability of exercise, the effects of regular exercise on bone health, and how one feels when they exercise to prevent osteoporosis. Calcium intake questions focus on the belief that adequate calcium intake reduces the risk for osteoporosis and broken bones. *Barriers to exercise *and* calcium intake* considers negative aspects of osteoporosis preventive behaviors. Barriers to exercise are evaluated through questions on mental and physical ability to exercise regularly, availability of time and exercise facilities, and family discouragement. Barriers to calcium intake questions assess the cost, preference for, ability to change dietary habits, cholesterol content, and digestive response to calcium intake. 

The final subscale of the OHBS, *health motivation*, evaluates the tendency to engage in healthy behaviors. Participants are asked to rate their diet, the importance of being healthy, desire for new health information, practice of obtaining regular health checkups, early diagnosis of health problems, and following recommendations. Possible scores for each subscale range from 6 to 30 points. Measures for the internal consistency of the OHBS are as follows: susceptibility, *α* = .82; seriousness, *α* = .71; benefits from exercise, *α* = .81; benefits from calcium intake, *α* = .80; barriers to exercise, *α* = .82; barriers to calcium intake, *α* = .74; health motivation, *α* = .73.

Self-efficacy refers to the individual's belief in their ability to successfully complete a specific task [13]. The Osteoporosis Self-Efficacy Scale (OSES) utilizes perceived susceptibility and seriousness, perceived barriers and benefits, health motivation, and self-confidence in one's ability to take actions needed to prevent osteoporosis to predict possible occurrence of health behaviors. The six items for assessing exercise self-efficacy measure confidence in beginning a new exercise program, changing exercise habits, giving the effort required to exercise, completing difficult exercises, time involved, and compliance with recommended exercises. Participants are asked to define their confidence level by placing a mark along a line connecting “not at all confident” and “very confident.” Scores are obtained by measuring the distance from the left anchor to the participants mark in millimeters. The range for each question is 0–100 millimeters. Individual scores for exercise self-efficacy are determined by adding the scores for the six items provided and dividing the total by six. The highest possible individual score would be 100. The OSES has a reliability measure of *α* = .90.

The Osteoporosis Knowledge Test (OKT) consists of two subscales: *OKT Exercise* consists of 16 items and *OKT Calcium* of 17 items. Nine items on the OKT assess the participants belief that a person is “more likely” to get osteoporosis, “less likely” to get osteoporosis, or “neutral” (the variable in question has nothing to do with getting osteoporosis). Examples of these characteristics include gender, race, diet, exercise, menopause, and steroid use. The remaining 15 questions are multiple choice and assess knowledge of exercise as an osteoporosis preventive behavior, and calcium sources and requirements. Responses on the OKT were scored as correct and incorrect. Individual score was determined by totaling the correct answers. Total possible score for *OKT Exerci*se was 16 points, for *OKT Calcium* was 17 points, and for OKT combined exercise and calcium (all questions on the OKT included) was 24 points. The OKT Exercise subscale has a reliability coefficient of *α* = .69 and the OKT Calcium subscale measured at *α* = .72.

All instruments in Chinese version were translated based on the processes of forward translation (into Chinese), back translation (into English), and comparison (between the original English version and the back translation version) [14]. The Osteoporosis Health Belief Scale had a Cronbach's Alpha of 0.68–0.84. Factor analysis showed seven subscales loading from 0.37–0.84 [15]. The Osteoporosis Self-efficacy Scale had a Cronbach's Alpha of 0.90–0.94 [16] and the Osteoporosis Knowledge Test scored 0.83–0.87 [16].

### 2.2. Methods

A convenience sample of undergraduate students attending The University of Mississippi *Withheld During Review*, United States (*n* = 408) and *Tianjin Medical University Withheld During Review*, Peoples Republic of China (*n* = 409) were administered the Osteoporosis Health Belief, Self-Efficacy, and Knowledge Tests. Participants in the USA were obtained through lecture classes that did not include health-related study (i.e., business, education, engineering, and liberal arts). Focus of study for the Chinese students included mechanical engineering, information technology, international business, science and engineering. None of the participants were studying medicine or nursing. For both universities, we emailed instructors asking for permission to survey their class. Surveys were administered at the beginning or end of class. Participants were informed about the nature of the study and that participation was voluntary. Institutional Review Board approval was received from both the US and Chinese Universities.

## 3. Results and Discussion

Analysis of the data was performed using SPSS 17.0. The mean age for the all participants was 19.38 ± 1.25 years. Mean age for US students was 19.49 ± 1.58 years. Chinese student mean age was 19.28 ± .86 years. Forty-two percent (*n* = 342) of the participants were male and 57% (*n* = 468) were female. For the US students only, 48% (*n* = 197) reported being male and 50% (*n* = 204) were female. The Chinese students reported a distribution of 35.5% (*n* = 145) as male and 64.5% (*n* = 264) being female. All of the Chinese students were classified as Asian. The US students reported being 74% (*n* = 301) White, 20% (*n* = 86) African-American, 1.5% (*n* = 6) Hispanic, 1% (*n* = 3) Asian, and 1.7% (*n* = 7) reported being classified as “other.” 

Scores for the Osteoporosis Health Belief Scale are reported in Table 1. Chi-square analysis showed a significant difference between the responses of the US students and the Chinese students for all of the subscales. For subscales where the *t*-test statistic was not significant but the chi-square statistic was (susceptibility and seriousness), *t*-tests were calculated for the individual questions that makeup the subscale to determine where the significant difference in distribution of the responses occurred (Table 2). For the susceptibility questions significance was found in only two of the six questions. US students reported a greater chance of getting osteoporosis and believed their body build increased the risk for the disease as compared to the Chinese students. However, these students were in agreement with the Chinese students when reporting the chance of actually becoming osteoporotic. Both groups disagreed that they had a good chance of getting osteoporosis. They both reported that they were less likely than the average person to get osteoporosis and that family history did not increase their risk for the disease. While US students have a greater awareness of the risk for getting osteoporosis than do the Chinese students, they, along with the Chinese students, do not believe that they will get the disease. 

For the seriousness subscale questions, significant differences were found in four of the six questions. The US students reported a greater belief that osteoporosis would cripple them, be very costly, and very serious. These students reported being more scared at the thought of having osteoporosis than did the Chinese students. However, the US and Chinese students agreed that having osteoporosis would change how they feel about themselves as well as getting depressed when they think about this disease. 

The US students reported significantly greater beliefs that exercise is preventive for osteoporosis and bone fractures, makes you feel better, and improves the body's appearance than did the Chinese students. The US students also had significantly greater beliefs that calcium intake can reduce the risk for osteoporosis and bone fractures. These students reported that adequate calcium intake would reduce their concern for getting osteoporosis. 

For the barriers of exercise subscale, the Chinese students reported having more difficulty in obtaining adequate exercise. These students reported not having a place to exercise, as well as greater discouragement from their family than did the US students. The Chinese students also felt that exercise would upset their daily routine, require the difficult task of forming a new habit, and be uncomfortable. The Chinese students had a significantly greater belief that they were not strong enough to exercise as compared to the US students.

The Chinese students also reported significantly greater barriers to calcium intake. These students were in greater agreement that calcium-rich foods cost too much, did not agree with their digestive tract, had too much cholesterol, or they just did not care for them than were the US students. The Chinese students also had a greater belief that increasing the amount of calcium-rich foods in their diet would be hard to do, and that they just do not care for these foods.

The health motivation subscale assessed beliefs regarding learning about health issues and staying healthy. US students had significantly greater responses to the items in this subscale. These students reported placing greater importance on keeping healthy than did the Chinese students. US students tend to look for new health-related information, have regular health checkups, and follow recommendations than do the Chinese students.

The total health belief score was significantly different between the US and Chinese students. Even though *t*-test analysis revealed no significant difference between the groups in perceived susceptibility or seriousness, minor differences did occur. The US students believed they had a greater chance of getting osteoporosis and that it would be a very serious, crippling condition than did the Chinese students. The US students also had a significantly greater perception of the benefits of exercise and calcium intake, as well as significantly less reported barriers to these preventive factors than did the Chinese students.

Scores for the Osteoporosis Knowledge Test are reported in Table 3. The scores for exercise knowledge and calcium knowledge subscales were low, as were the total osteoporosis knowledge scores. The US students answered 56.5% of the questions for exercise knowledge correctly, while the Chinese students had only 45.4% correct. The US mean score was significantly higher than that of the Chinese students (*t* = 8.30, *P* < .05). For calcium knowledge related to osteoporosis US students answered 57.2% of the questions correctly and Chinese students 42.5%. There was also a significant difference between the mean calcium knowledge scores (*t* = 11.34, *P* < .05). Total osteoporosis knowledge was also significantly greater for the US students who answered 60.5% of the questions correctly as compared to the Chinese students who got 49.2% correct (*t* = 9.31, *P* < .05). 

Chi-square analysis of the individual knowledge questions revealed significant differences (*P* < .05) for all but four of the 24 questions. More US students answered correctly when identifying dairy intake, menopausal status, genetics, race, and exercise as risk factors for osteoporosis than did Chinese students (Table 4). No significant difference was found between the groups when asked how having the ovaries surgically removed affects the risk for osteoporosis (*X* ^2^ = 1.16, *P* > .05). 

Seventy-four percent of the US students knew that aerobic dancing and jogging or running were good activities for reducing the risk of osteoporosis. Only about 57% of the Chinese students correctly identified these. Almost 45% of the US and 40% of the Chinese students selected yoga over bicycling as the best activity for reducing the risk of osteoporosis. Swimming was also selected as a preventive activity (US = 57%, China = 43%) over walking briskly. Interestingly, approximately 10% of the Chinese students identified house work, such as washing dishes or cooking as a better preventive activity than walking briskly or bicycling.

When identifying good sources of calcium, the US students answered correctly significantly more often than the Chinese students, however, the number of students selecting correct choices was low (Table 4). Almost 38% of the Chinese students identified chicken as a better source of calcium than broccoli (23.5% of the US students also chose chicken). Approximately 20% of the Chinese student selected cabbage as a better calcium source than yogurt. Other foods the Chinese students selected as good sources of calcium include grapefruit (33.5%) and radishes (26%). Significantly more Chinese student's correctly selected canned sardines as a good calcium source than did US students. Thirty percent of US students and 31% of Chinese selected corn over sardines as a good source of calcium.

Both groups of students did poorly when asked to select the recommended daily calcium intake for an adult. Only 9.3% of US students and 10.5% of the Chinese students were able to identify 800 mg* or more daily* as the correct answer. 

When assessing differences between male and female population for both USA and China, the perception of susceptibility to getting osteoporosis was the only significant variable found (*t* = −5.06, *P* = .000). Female students had a greater perception of susceptibility than their male counterparts (female mean = 13.88, SD = 4.53, male mean = 12.24, SD = 4.41). 

Significant differences between the male and female students from the USA were found in several of the variables (Table 5). The students from China revealed no significant differences between the males and females for all variables.

The mean osteoporosis self-efficacy score for all the students was 68.74 ± 21.76 (from a possible score of 100). A significant difference was found between the mean US and Chinese students reported self-efficacy measures (73.7 ± 21.5 and 63.8 ± 20.89, resp., *t* = 6.64, *P* < .05). The US students reported more confidence in beginning an exercise program, changing exercise habits, exerting the effort required to exercise, attempting difficult exercises, exercising for the appropriate length of time, and doing exercises that will help reduce the risk of osteoporosis. 

A limitation of this study is that the sample selections were limited to college students and therefore may not be representative of osteoporosis knowledge, perceptions, and self-efficacy across China or the USA. Another limitation of the instrument used is that confounding variables such as family history of osteoporosis or presence of an illness requiring medication were not assessed.

## 4. Discussion

In this study we examined osteoporosis knowledge, beliefs, and self-efficacy in college students in China and the USA. Students from the USA were found to have greater knowledge of osteoporosis and of the influence that exercise and calcium have on this disease. The US students also had stronger osteoporosis health beliefs for benefits and fewer barriers to exercise and calcium intake. The US students had significantly greater total health belief scores; however, their perceptions of susceptibility to and seriousness of osteoporosis were similar to those of the Chinese students. The students agreed that it is unlikely that they will get osteoporosis and that family history does not influence their risk. The perception of not being at risk for chronic diseases is not uncommon in college age students. Increasing knowledge of disease and risk factors among this population is warranted. Both groups of students were also in agreement that having osteoporosis would not change how they feel about themselves and that thinking about osteoporosis does not depress them. The US students reported significantly greater self-efficacy for exercise in the prevention of osteoporosis when compared to the Chinese students. This may be a cultural difference influencing the perception and availability of exercise. 

The finding that US college aged students have greater knowledge regarding osteoporosis is of interest; however, the mean score was only 60% indicating that both Chinese and US students have an inadequate knowledge of osteoporosis. Efforts to increase awareness of osteoporosis are important at this age. Many in this population may not have reached peak bone mass and with adequate knowledge, they may be able to increase the level at which their bone mass may ultimately reach. College campuses need to be assessed for the best venues for providing osteoporosis information and programming to this population.

Even though the US participants had greater knowledge of osteoporosis, and generally a greater perception of the disease, they, along with the Chinese students were not concerned with the possibility of getting the disease. It is often found that knowledge and perceptions are not reflected in behaviors. Assessing preventive behaviors such as calcium intake and exercise habits is needed to determine if these perceptions are reflected in behaviors which may increase the risk for low BMD.

Barriers to exercise and calcium intake were greater for the Chinese students. This may be due to cultural differences in dietary and physical activity habits. The typical Chinese diet consists of cereals and vegetables with a minimal intake of animal products, limiting calcium availability [17]. Zhai et al. [18] reported that between 1989 and 2004 calcium intake in China did not increase but remained low. Individuals living in the urban areas of China took in an estimated 430 mg of calcium daily, while those living in rural areas consumed only 380 mg per day. Knowledge of the role of calcium in bone health, and obtainable sources of calcium, is needed to influence greater calcium intakes in the Chinese population. The US students reported fewer barriers to calcium intake; however, it would be of interest to assess calcium intake behaviors to determine if the lack of barriers is reflected in the intake behavior (data from NHANES 1999–2002 found that US males, aged 20–39 years, consumed approximately 1,054 mg daily and females consumed 800 mg per day [19]). College campuses in the USA typically have good access to foods, including dairy products. Emphasizing appropriate serving sizes, and the need for calcium in the diet should be encouraged on college campuses.

The Chinese student's greater sense of barriers to exercise reflects their belief that there is no encouragement or place for them to exercise. They also report that exercise would upset their daily routine making it a difficult habit to conform to. This may be a cultural aspect which requires further investigation. 

The perception of susceptibility to osteoporosis was found to be the only significant difference between the genders when assessing all of the student participants. This subscale was also significant between the US but not the Chinese genders. Our assertion is that media presentation of osteoporosis is more commonly seen in the USA compared to China. The US media frequently presents osteoporosis as a female disease which may influence the finding that females in the USA have a greater sense of susceptibility than do the males. The influence of the media may also explain the significant differences in total knowledge of osteoporosis, health motivation, and total health belief score between the US genders. To improve US male perception and both Chinese female and male perception of the risks for osteoporosis, the media needs to expand their influence to affecting both males and females in both countries. Further studies into the influence of media on osteoporosis knowledge and perceptions within and between genders is warranted.

Exercise self-efficacy differed significantly between the two groups and may possibly explain the increase in barriers to exercise perceived by the Chinese students. Pearson Correlation tests of exercise self-efficacy and barriers of exercise revealed low, but significant negative relationships. Self-efficacy had a stronger relationship with exercise barriers for the US students (*r* = −.37, *P* < .05) than for the Chinese students (*r* = −.24, *P* < .05). As previously reported, the US students had greater Total Knowledge scores which could explain the significantly lower perception of barriers to exercise.

In conclusion, the findings from this study suggest that college students in the USA are more informed about osteoporosis and have greater perceptions regarding the influence of exercise and calcium intake on this disease when compared to Chinese students. However, the US and Chinese students have a similar low perception of their susceptibility to or the seriousness of osteoporosis. It is not uncommon for college students to have low disease susceptibility or seriousness of a chronic disease. This needs to be addressed as an international factor when combating osteoporosis. There are many differences in lifestyles between the USA and China, as well as within the USA and China alone, which may affect predictors of osteoporosis. Differences in dietary intake and exercise habits may influence risk factors for this disease. This should also be taken into consideration when developing strategies and prevention programs for osteoporosis. The present study offered us the opportunity to compare the osteoporosis knowledge and perceptions of two cultures. This study, however, did not allow for in-depth insight into behaviors of the participants. Future studies need to assess preventive behaviors (i.e., exercise and calcium intake) in these students. Such studies may be more beneficial if conducted separately, as the focus can be on the cultural influences of behavior.

## Figures and Tables

**Table 1:** Osteoporosis Health Belief Scale scores of college- aged students from China and the USA.

Subscale (6–30 possible points)	All participants *n* = 774 Mean ± SD	USA *n* = 408 Mean ± SD	China *n* = 409 Mean ± SD	*t*-test	*P-*value for *t*-test	X^2^	*P-*value for X^2^
Susceptibility	13.16 ± 4.55	13.44 ± 4.58	12.88 ± 4.50	1.72	.087	47.43*	.001
Seriousness	14.39 ± 3.31	14.44 ± 3.50	14.35 ± 3.11	0.37	.712	30.83*	.030
Benefits of exercise	22.59 ± 4.50	24.07 ± 3.81	21.09 ± 4.66	9.89*	.000	121.6*	.000
Benefits of calcium	20.77 ± 4.51	23.17 ± 3.89	18.36 ± 3.77	17.81*	.000	278.22*	.000
Barriers of exercise	13.36 ± 4.76	11.75 ± 4.55	14.96 ± 4.41	−10.07*	.000	110.07*	.000
Barriers of calcium	14.39 ± 4.81	13.04 ± 4.63	15.74 ± 4.60	−8.26*	.000	93.06*	.000
Health motivation	20.97 ± 4.32	21.42 ± 3.95	20.51 ± 4.62	2.99*	.003	42.04*	.009
Health belief total score (42–210 possible points)	122.17 ± 14.91	124.68 ± 13.12	119.55 ± 16.18	4.68*	.000	115.33*	.003

*Significant at *α* = .05.

**Table 2:** Responses for Susceptibility and Seriousness subscale questions of college-aged students from China and the USA.

(5 possible points)	USA *n* = 408 Mean ± SD	China *n* = 409 Mean ± SD	*t*-test	*P*-value
(1) Your chances of getting osteoporosis are high.	2.39 ± .94	2.03 ± .91	5.60*	.000
(2) Because of your body build, you are more likely to develop osteoporosis.	2.41 ± .98	2.11 ± .88	4.51*	.000
(3) It is extremely likely that you will get osteoporosis.	2.08 ± .86	2.08 ± .91	−.02	.983
(4) There is a good chance that you will get osteoporosis.	2.27 ± .89	3.27 ± 1.0	−1.51	.131
(5) You are more likely than the average person to get osteoporosis.	2.19 ± .93	2.17 ± 1.02	.24	.807
(6) Your family history makes it more likely that you get osteoporosis.	2.13 ± 1.04	2.14 ± 1.08	−.23	.818
(7) The thought of having osteoporosis scares you.	3.19 ± 1.17	2.97 ± 1.08	2.68*	.007
(8) If you had osteoporosis you would be crippled.	2.51 ± .97	2.29 ± 1.12	2.96*	.003
(9) Your feelings about yourself would change if you got osteoporosis.	2.84 ± 1.13	2.70 ± 1.35	1.78	.075
(10) It would be very costly if you got osteoporosis.	3.37 ± 1.01	2.87 ± 1.14	6.93*	.000
(11) When you think about osteoporosis you get depressed.	2.45 ± 1.05	2.48 ± 1.09	−.44	.661
(12) It would be very serious if you got osteoporosis.	3.54 ± 1.04	2.64 ± 1.10	11.89*	.000

*Significant at *α* = .05.

**Table 3:** Osteoporosis Knowledge scores of college- aged students from China and the USA.

Subscale	All participants *n* = 774 Mean ± SD	USA *n* = 408 Mean ± SD	China *n* = 409 Mean ± SD	*t*-test
Total knowledge score (24 possible points)	13.7 ± 4.18	14.52 ± 4.16	11.82 ± 3.76	9.31*
Exercise knowledge score (16 possible points)	8.16 ± 3.06	9.04 ± 3.17	7.27 ± 2.67	8.30*
Calcium knowledge score (17 possible points)	8 .47 ± 3.29	9.73 ± 3.35	7.23 ± 2.71	11.34*

*Significant at *α* = .05.

**Table 4:** Responses to the Osteoporosis Knowledge Tests of college aged students from China and the USA.

Question, *correct response in italics *	USA *n* = 408 % correct	China *n* = 409 % correct	X^2^
(1) Eating a diet low in milk products, *more likely to get osteoporosis *	62.9	47.7	19.12*
(2) Being menopausal, *more likely to get osteoporosis *	44.3	21.1	49.72*
(3) Having big bones, *less likely to get osteoporosis *	25.1	37.8	14.98*
(4) Eating a diet high in dark green leafy vegetables, *less likely to get osteoporosis *	59.5	42.5	23.29*
(5) Have a mother or grandmother who has osteoporosis, *more likely to get osteoporosis *	67.4	24.4	150.03*
(6) Being a white woman with fair skin, *more likely to get osteoporosis *	27.6	12.4	29.28*
(7) Having ovaries surgically removed, *more likely to get osteoporosis *	14.1	11.6	1.16
(8) Taking cortisone for long time, *more likely to get osteoporosis *	48.4	24.6	49.40*
(9) Exercising on a regular basis, *less likely to get osteoporosis *	74.1	45.0	71.85*
(10) Which exercise is best to reduce risk for osteoporosis, *walking briskly *	23.5	37.4	18.53*
(11) Which exercise is best to reduce risk for osteoporosis, *bicycling *	35.4	40.5	2.23
(12) How many day a week do you need to exercise to strengthen bones, *3 or more days *	81.2	48.0	101.92*
(13) Least amount of time to exercise on each occasion to strengthen bones, *20–30 minutes *	73.4	58.6	19.76*
(14) Exercise must be hard enough to make breathing, *much faster, but talking is possible *	47.4	58.3	1.45
(15) Which exercise is best to reduce chance of getting osteoporosis, *jogging or running *	74.0	63.5	10.32*
(16) Which exercise is best to reduce chance of getting osteoporosis, *aerobic dancing *	74.4	51.5	25.65*
(17) Which is a good source of calcium, *cheese *	86.5	69.9	32.65*
(18) Which is a good source of calcium, *canned sardines *	17.5	44.99	71.67*
(19) Which is a good source of calcium, *broccoli *	44.7	33.0	11.69*
(20) Which is a good source of calcium, *yogurt *	87.6	60.9	76.20*
(21) Which is a good source of calcium, *ice cream *	59.2	24.7	99.34*
(22) What is recommended amount of calcium for an adult, 800 mg* or more daily *	9.33	10.5	.32
(23) How much milk must adult drink to meet recommended intake, *2 or more glasses daily *	45.21	19.85	59.70*
(24) Which is best reason for taking calcium supplement, *not get enough calcium from diet *	72.51	47.68	88.96*

*Significant at *α* = .05.

**Table 5:** Comparison of Osteoporosis Knowledge Test and Health Belief Scale scores of male and female college aged students from the USA.

Subscale (6–30 possible points)	Female Mean ± SD (*n*)	Male Mean ± SD (*n*)	*t*-test	*P* value
Total knowledge	15.12 ± 3.97 (184)	14.02 ± 4.17 (185)	−2.59*	.010
Exercise knowledge	9.40 ± 3.05 (186)	8.75 ± 3.21 (189)	−2.03*	.043
Calcium knowledge	10.14 ± 3.23 (187)	9.42 ± 3.30 (187)	−2.12*	.035
Susceptibility	14.86 ± 4.44 (200)	12.08 ± 4.31 (195)	−6.32*	.000
Seriousness	14.52 ± 3.59 (196)	14.35 ± 4.43 (196)	−0.46	.646
Benefits of exercise	24.22 ± 3.84 (203)	23.95 ± 3.55 (190)	−0.71	.480
Benefits of calcium	23.21 ± 3.87 (202)	23.18 ± 3.64 (191)	−0.06	.948
Barriers of exercise	11.52 ± 4.41 (201)	12.07 ± 4.68 (188)	1.20	.232
Barriers of calcium	12.68 ± 4.57 (200)	13.46 ± 4.62 (191)	1.68	.094
Health motivation	21.80 ± 3.75 (202)	21.01 ± 3.91(190)	−2.04*	.042
Health belief total score, (42–210 possible points)	126.48 ± 12.66 (187)	123.38 ± 12.00 (176)	−2.39*	.017

*Significant at *α* = .05.
